# Supplementary material for: Exploring Southern Ecuador’s Traditional Medicine: Biological Screening of Plant Extracts and Metabolites
Source: Plants (Basel). 2024 May 20;13(10):1422. doi: 10.3390/plants13101422 (PMC11124848; doi:10.3390/plants13101422)
Supplement: Supplementary file 1 [file plants-13-01422-s001.zip › plants-2931832-supplementary.pdf]

# SUPPLEMENTARY INFORMATION

# Exploring Southern Ecuador's Traditional Medicine: Biological Screening of Plants Extracts and Metabolites

**Nicole Bec [1], Christian Larroque [2,3] and Chabaco Armijos [3,*]**

**Figure S1.** Microtubules network upon incubation with extracts Page 2
**Figure S2.** Centrosomal gTub localization upon incubation with the extracts Page 3
**Figure S3.** Nuclear gH2aX foci formation upon incubation with the extracts. Page 4
**Figure S4.** Cadherin localization upon incubation with extracts Page 5

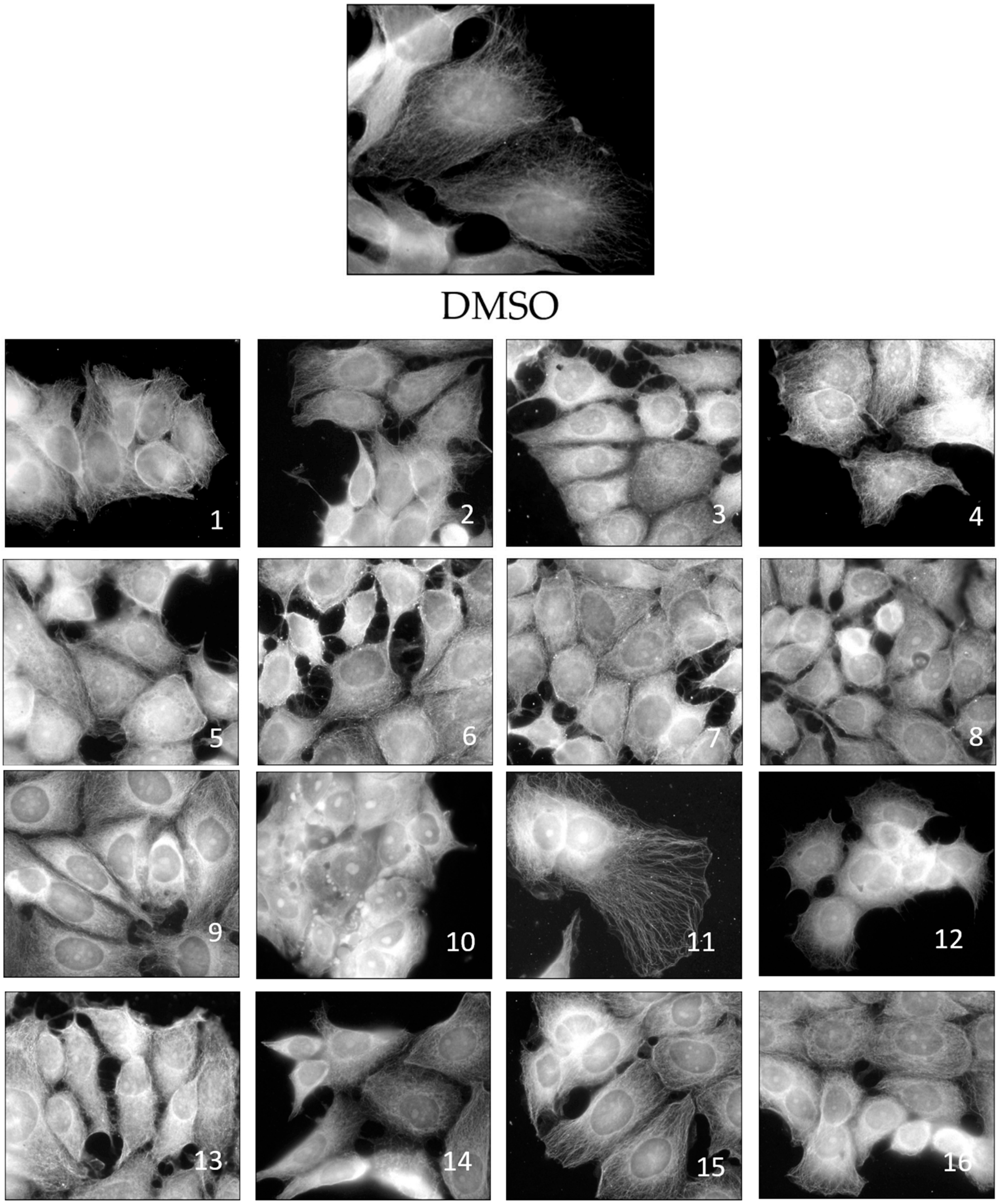


**Figure S1.** Microtubules network upon incubation with extracts.

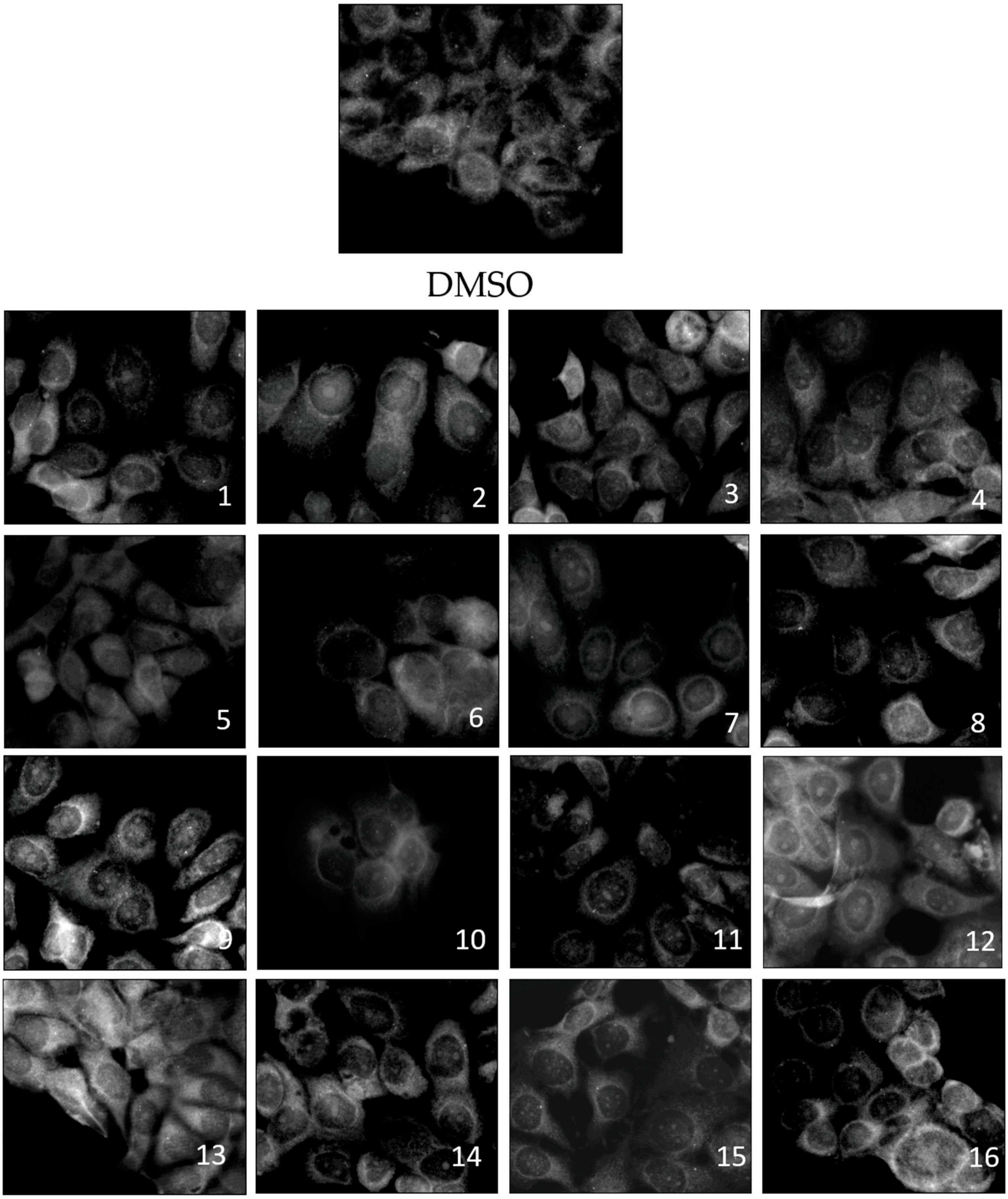


**Figure S2.** Centrosomal gTub localization upon incubation with the extracts.

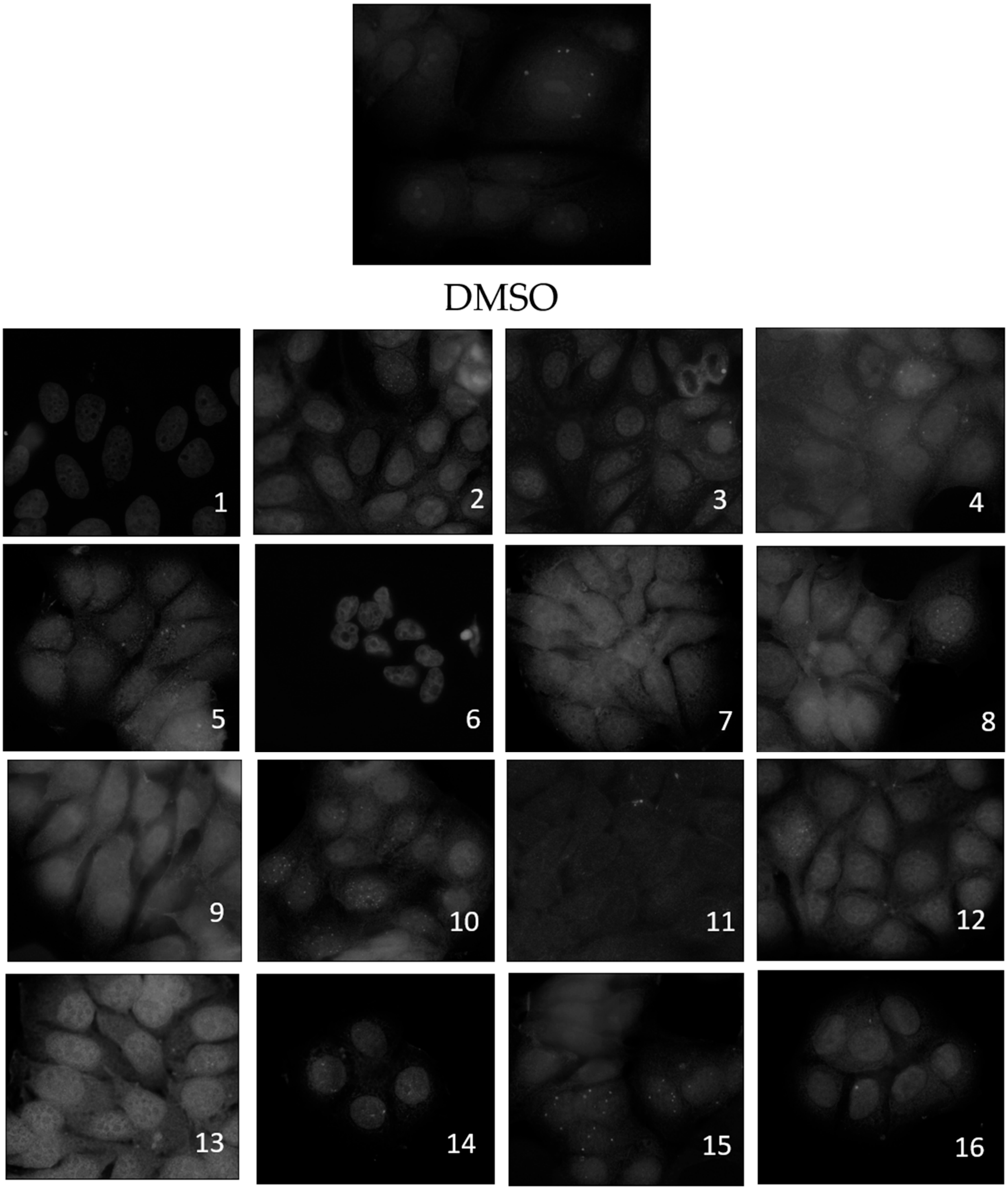


**Figure S3.** Nuclear gH2aX foci formation upon incubation with the extracts.

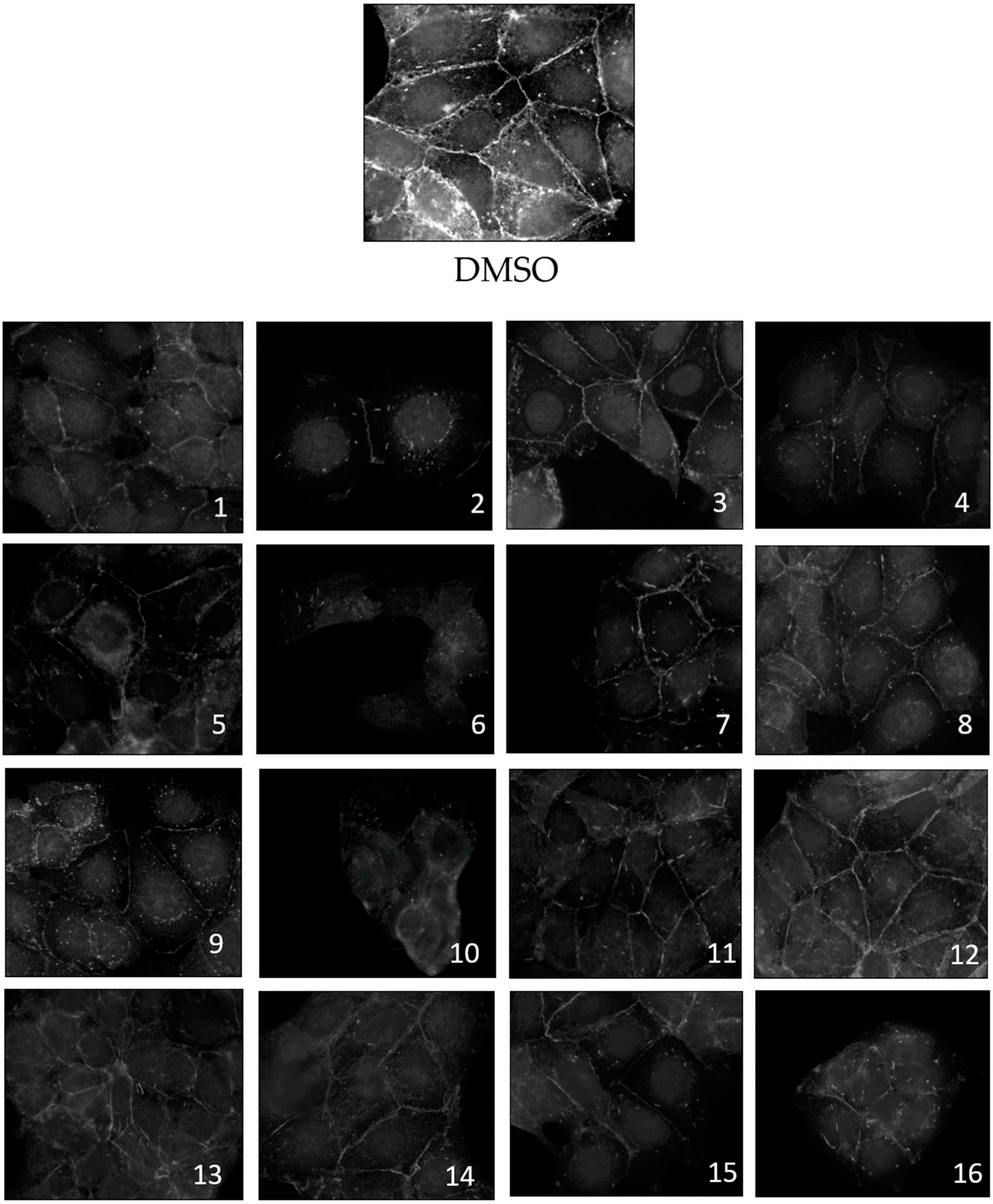


**Figure S4.** Cadherin localization upon incubation with extracts.
